# Relationship between Dietary Fatty Acid Intake with Nonalcoholic Fatty Liver Disease and Liver Fibrosis in People with HIV

**DOI:** 10.3390/nu13103462

**Published:** 2021-09-29

**Authors:** Cristiane Fonseca de Almeida, Paula Simplicio da Silva, Claudia Santos de Aguiar Cardoso, Nathalia Gorni Moreira, Julliana Cormack Antunes, Michelle Morata de Andrade, Julio Silva, Marina Campos Araujo, Wilza Arantes Ferreira Peres, Pedro Emmanuel Alvarenga Americano do Brasil, Ronaldo Ismerio Moreira, Sandra W. Cardoso, Valdilea G. Veloso, Beatriz Grinsztejn, Patricia Dias de Brito, Hugo Perazzo

**Affiliations:** 1 Grupo de Pesquisa Clínica em Nutrição e Doenças Infecciosas (GPClin_Nut), Instituto Nacional de Infectologia Evandro Chagas—Fundação Oswaldo Cruz (FIOCRUZ), 21040-360 Rio de Janeiro, Brazil; cristiane.almeida@ini.fiocruz.br (C.F.d.A.); paula.simplicio@ini.fiocruz.br (P.S.d.S.); claudia.cardoso@ini.fiocruz.br (C.S.d.A.C.); julliana.cormack@ini.fiocruz.br (J.C.A.); pedro.brasil@ini.fiocruz.br (P.E.A.A.d.B.); patricia.brito@ini.fiocruz.br (P.D.d.B.); 2 Serviço de Nutrição (SENUT), Instituto Nacional de Infectologia Evandro Chagas—Fundação Oswaldo Cruz (FIOCRUZ), 21040-360 Rio de Janeiro, Brazil; nathalia_gorni@hotmail.com; 3 Plataforma de Pesquisa Clínica, Instituto Nacional de Infectologia Evandro Chagas—Fundação Oswaldo Cruz (FIOCRUZ), 21040-360 Rio de Janeiro, Brazil; Michelle.morata@ini.fiocruz.br (M.M.d.A.); julio.lima@ini.fiocruz.br (J.S.); 4 Escola Nacional de Saúde Pública—Fundação Oswaldo Cruz (FIOCRUZ), 21031-210 Rio de Janeiro, Brazil; mcamposaraujo@gmail.com; 5 Instituto de Nutrição Josué de Castro, Universidade Federal do Rio de Janeiro (UFRJ), 21941-902 Rio de Janeiro, Brazil; wilza@nutricao.ufrj.br; 6 Laboratório de Pesquisa em Imunização e Vigilância em Saúde, Instituto Nacional de Infectologia Evandro Chagas—Fundação Oswaldo Cruz (FIOCRUZ), 21040-360 Rio de Janeiro, Brazil; 7 Laboratório de Pesquisa Clínica em DST/AIDS (LAPCLIN-AIDS), Instituto Nacional de Infectologia Evandro Chagas—Fundação Oswaldo Cruz (FIOCRUZ), 21040-360 Rio de Janeiro, Brazil; ronaldo.ismerio@ini.fiocruz.br (R.I.M.); sandra.wagner@ini.fiocruz.br (S.W.C.); valdilea.veloso@ini.fiocruz.br (V.G.V.); beatriz.grinsztejn@gmail.com (B.G.)

**Keywords:** dietary fats, liver fibrosis, NAFLD, HIV infection

## Abstract

We aimed to evaluate the relationship between food intake of lipids with nonalcoholic fatty liver disease (NAFLD) and/or liver fibrosis in people living with HIV/AIDS (PLWHA). In this cross-sectional study, transient elastography was used to detect the presence of NAFLD and/or liver fibrosis. The dietary intake of fats and fatty acids (FA) were assessed by two 24 h dietary recalls (24-HDR) (*n* = 451). Multivariate logistic regression models were performed. Participants with higher intake of total fat were associated with higher odds for NAFLD compared to those with lower consumption [adjusted odds ratio (aOR) = 1.91 (95% confidence interval (95% CI) 1.06–3.44)]. Furthermore, participants with intermediate intake of n6-PUFA (n6-poly-unsaturated FA) and lauric FA had lower odds for NAFLD, respectively aOR = 0.54 (95% CI 0.3–0.98) and aOR = 0.42 (95% CI 0.22–0.78). Additionally, a higher intake of myristoleic FA (fourth quartile) was a significant protective factor for NAFLD [aOR = 0.56 (95% CI 0.32–0.99)]. Participants with higher intake of lauric FA [0.38 (95% CI 0.18–0.80)], myristic FA [0.38 (0.17–0.89)], palmitoleic FA [0.40 (0.19–0.82)] and oleic FA [0.35 (0.16–0.79)] had positively less odds of having liver fibrosis. On the other hand, higher intake of n-6 PUFA was significantly associated with fibrosis [aOR = 2.45 (95% CI 1.12–5.32)]. Dietary assessment of total fat and FA should be incorporated into HIV care as a tool for preventing NAFLD and fibrosis in PLWHA.

## 1. Introduction

Globally, 38 million people have been living with the human immunodeficiency virus (HIV) [1]. The use of early combined antiretroviral therapy (c-ART) has been decreasing the incidence of opportunistic diseases and increased the life expectancy in people living with HIV/AIDS (PLWHA) [2]. In contrast, the prevalence of non-communicable diseases has been dramatically increasing in PLWHA in the last decade [3]. Non-alcoholic fatty liver disease (NAFLD) is characterized by abnormal accumulation of fat in the liver in the absence of abusive alcohol intake. Clinical presentation of NAFLD can range from simple steatosis to nonalcoholic steatohepatitis (NASH) that can progress to cirrhosis and its complications, such as hepatocellular carcinoma. The presence of advanced liver fibrosis is the main predictor of mortality in individuals with NAFLD [4]. Several studies have been reporting the burden of NAFLD and/or liver fibrosis in PLWHA [5,6,7].

Dietary habits seem to play an important role in the pathogenesis of NAFLD. The Western diet has been associated with high levels of inflammatory cytokines [8] and a higher prevalence of NAFLD in the general population [9]. On the other hand, the Mediterranean diet can reduce fatty liver and improve insulin resistance status [10]. However, the influence of specific nutrients has not been fully elucidated [11]. Among dietary factors, total fat intake and analysis of the specific subtype of fatty acid (FA) intake might be relevant due their functional and metabolic distinct effects [12]. This might be reinforced because the dietary FA composition impacts liver metabolism, leading to triglyceride accumulation in the liver tissue [13]. However, this relationship has not been completely studied, especially in PLWHA. Studies conducted in Brazil showed that patients with NAFLD had high energy and lipid consumption [14]. Additionally, studies have demonstrated that PLWHA presented more likely an unhealthy food intake pattern [15]. However, the relationship between dietary fat intake and their subtype of fatty acid with NAFLD and liver fibrosis has not been studied in PLWHA. Therefore, the aim of this study was to evaluate the relationship between dietary fatty acid intake and NAFLD and/or the presence of liver fibrosis in HIV mono-infected individuals.

## 2. Materials and Methods

### 2.1. Study Design and Participants

This cross-sectional study analyzed data collected at the baseline visit from the longitudinal PROSPEC-HIV study (NCT02542020) that has been conducted at Evandro Chagas National Institute of Infectious Diseases (INI/FIOCRUZ, Rio de Janeiro, Brazil) [16]. All participants with HIV infection enrolled in the PROSPEC-HIV study from June 2015 to January 2019 were eligible for this analysis. Participants with viral hepatitis co-infection defined by positive HCV-antibody or positive HBsAg; excessive alcohol consumption defined by the Alcohol Use Disorders Identification Test (AUDIT) score ≥8 [17]; use of lipids supplements or missing laboratory/inconsistent data on dietary assessment were excluded. This study was approved by the Ethical Committee from INI/FIOCRUZ (IRB 32889514.4.0000.5262). All participants signed an informed consent upon enrollment in the PROSPEC-HIV study.

### 2.2. Clinical Assessment and HIV Infection History

Clinical records collected at baseline visit of PROSPEC HIV study included age, sex at birth, self-reported skin-color [18], years of study and presence of co-morbidities. Dyslipidemia, hypertension, type-2 diabetes and metabolic syndrome were defined according to the International Diabetes Federation [19]. Anthropometric measures, such as weight, height and waist circumference were measured by trained research assistants. Participants were considered as lean, overweight and obese if body mass index (BMI) < 25 Kg/m^2^, BMI = 25 to 29.99 Kg/m^2^ and BMI ≥ 30 Kg/m^2^, respectively [20]. A bioelectrical impedance analyzer (Biodynamics^®^ 450, Sao Paulo, Brazil) with 4-electrode (hand-feet) and frequency of 50 kHz was used to assess body fat percentage. All bioelectrical impedances were performed by a single operator in fasted participants in supine position [21]. The following data were available at the INI/FIOCRUZ HIV clinical cohort: (i) date of first positive HIV antibody test; (ii) date of initiation of any antiretroviral drug; (iii) dates of start and end of combined antiretroviral therapy (c-ART) and (iv) CD4^+^ T-lymphocyte count and HIV viral load from the closely day of clinical visit.

### 2.3. Laboratory Tests and Transient Elastography

Blood tests were performed after an overnight fasting and analyzed in a centralized laboratory using an analyzer Dimension-RxL-Max (Siemens Healthcare Diagnostic, Hoffman Estates, IL, USA). Liver tests, such as alkaline phosphatase, alanine aminotransferase (ALT), aspartate aminotransferase (AST) and gamma-glutamyltransferase (GGT) were measured using an enzymatic assay. Glucose was measured using the hexokinase method; total and high-density lipoprotein (HDL) cholesterol and triglycerides were determined using enzymatic methods. Low-density lipoprotein (LDL) cholesterol was calculated using the Friedewald equation [16]. Insulin was determined using chemiluminescent immunoassay (CLIA) and the Homeostatic Model Assessment for Insulin Resistance (HOMA-IR) was calculated by the formula: [fasting insulin (mIU/L) × fasting glucose (mg/dL)]/405 [22].

Transient elastography (TE) by FibroScan (EchoSens, Paris, France) was performed by a single experienced (>2000 examinations) operator (HP) to detect the presence of NAFLD and/or liver fibrosis following a previously described validated procedure [16]. The results defined as a median of 10 valid measures and expressed in kPa were considered as reliable for analysis if the following criteria had been met: (1) at least 10 valid measurements; (2) an interquartile range (IQR) lower than 30% of the median of liver stiffness measurement (LSM) for fibrosis or Controlled Attenuation Parameter (CAP) for steatosis; and (3) a success rate of more than 60%. The results of XL probe were considered in participants with unreliable TE exams with the M probe. NAFLD was defined by CAP ≥ 248 dB/m [23]. Presence of significant liver fibrosis (METAVIR stage F ≥ 2) was defined by LSM ≥ 7.1 kPa or ≥6.2 kPa with M or XL probe, respectively [24].

### 2.4. Dietary Data

The dietary intake of macronutrients, fat subtypes and FA were assessed by the 24 h dietary recall (24-HDR) method. Briefly, a nutritionist investigator requested the participants to self-report all foods and beverages consumed through the last 24 h. These reports must include details of food preparation and type of oil or fat used, as well as amount of food consumed in household measurements [25]. The 24-HDR was applied using the Automated Multiple-Pass method to structure the interview and to increase the accuracy of the report, minimizing any memory bias [26]. In addition, the 24-HDR was performed in two non-consecutive days: a face-to-face interview during the clinical visit and a remote interview by telephone a few days later. Data of each food and/or beverage item reported by the participant were converted to milligrams/grams and/or milliliters/liters, and these data were entered into a nutritional analysis software (Diet Win Professional Plus 3.0^®^ package software) that uses the Brazilian nutrient database, known as TACO (“Tabela Brasileira de Composição de Alimentos”). The implausibility in self-report intake was verified when individual report less than three foods items. In addition, the 24-HDR that had extreme values (outliers) of caloric intake were reviewed by boxplot graphs to evaluate possible inconsistent data.

The statistical modeling technique Multiple Source Method (MSM) was used to estimate the usual intake of nutrients of participants and to correct the intrapersonal dietary variability (https://msm.dife.de, accessed on 7 July 2019). The use of this approach to correct this variability avoids the need of multiple dietary interviews to estimate individual dietary intake [27]. Nutrients were adjusted by energy density method (the ratio between usual nutrient intake and total usual energy intake) expressed as a percentage to evaluate the relative contribution of these nutrients to the diet [28]. Fiber intake was calculated per 1000 kcal using the following formula: total fiber (g) × 1000 kcal/total energy intake.

### 2.5. Statistical Analysis

**Figure 1:**
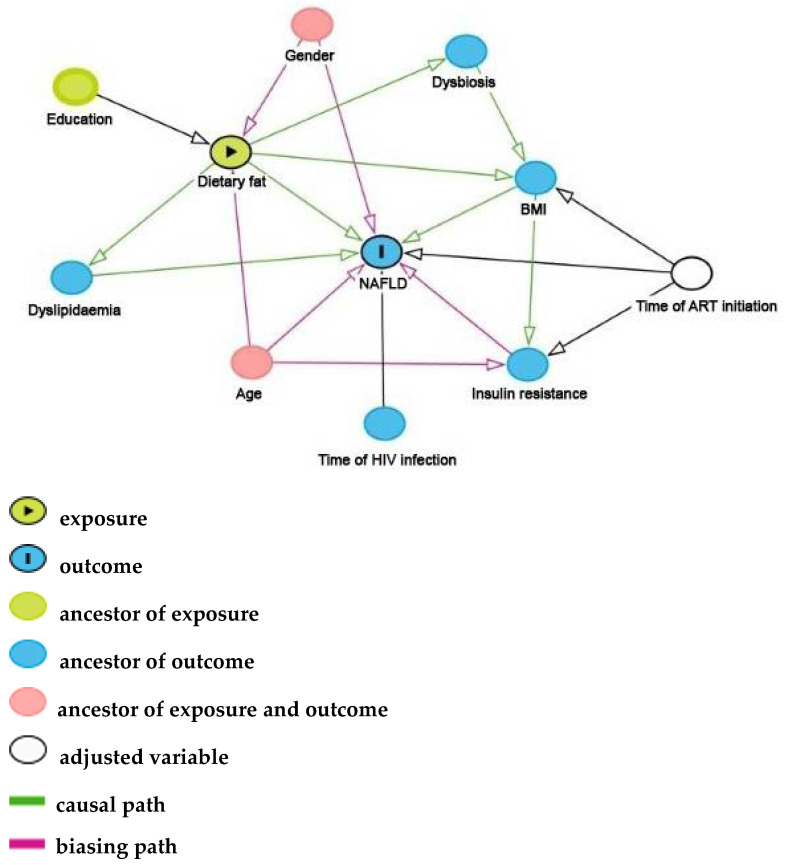
Direct Acyclic Graph for association of dietary fats and fatty acids with liver fibrosis and NAFLD in HIV patients, Rio de Janeiro, Brazil.

Categorical variables were reported as absolute (*n*) and relative frequency (%) and continuous variables as median and interquartile range (IQR). We used Chi-square and Mann—Whitney tests to compare proportions and medians, respectively. All nutrients were analyzed in proportion of energy intake (E%). Direct Acyclic Graphs (DAGs) were created with assumptions on the relationship among co-variables and outcomes (NAFLD or fibrosis) using the DAGitty as a browser-based environment (http://www.dagitty.net/, accessed on 6 December 2019) (Figure 1). DAG, as illustrated in Figure 1, is a theoretical model described through a graph that permits qualitative and visual assessment of confounding factors. These DAGs supported our decision about the most parsimonious models for NAFLD and fibrosis to avoid collinearity and confounding. Logistic multivariate models considered occurrence of NAFLD and liver fibrosis as outcomes, each nutrient alone (in quartiles) as independent variables (assuming quartile 1, lowest consumption as the reference), and age, sex and duration of c-ART as confounders as well as usual energy intake (kcal) to minimize the underreporting of the food intake method. Statistical analyses were performed using R version 3.6.3 and considering *p*-values < 0.05 as statistically significant.

## 3. Results

### 3.1. Study Characteristics

**Figure 2:**
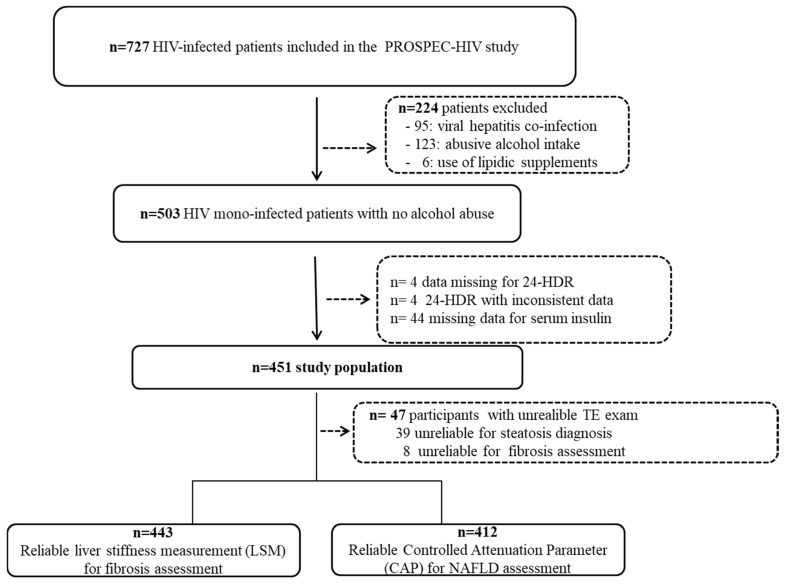
Flow chart of patient recruitment, Rio de Janeiro, Brazil.

A total of 727 participants with HIV infection were included in the PROSPEC-HIV study from June 2015 to January 2019. For this analysis, participants were excluded due to viral hepatitis coinfection (*n* = 95), abusive alcohol intake (*n* = 123), use of lipid supplement (*n* = 6), 24-HDR with missing (*n* = 4) or inconsistent dietary data (*n* = 4) or missing data of serum insulin (*n* = 44). The flowchart of the study population is depicted in Figure 2.

A total of 451 participants [60.3 female, median age of 45 (IQR, 36–53) years, 33.9% with metabolic syndrome, median BMI = 25 (IQR, 23–29) Kg/m^2^, 96.7% under c-ART during a median time of 7 (IQR, 4–14) years] were included in this analysis. Table 1 describes clinical and demographic characteristics of the participants. CAP and LSM values were unreliable with M and XL probes in 9% (*n* = 39) and 2% (*n* = 8) of participants, respectively. Therefore, the association of lipid dietary intake with NAFLD and significant fibrosis was assessed in 412 and 443 HIV mono-infected participants, respectively. The prevalence of NAFLD and significant fibrosis were 37% (95% CI, 32–41) [*n* = 152] and 16% (95% CI, 12–20) [*n* = 72], respectively. Supplementary Tables S1 and S2 describe the socio-demographic and clinical characteristics of participants with NAFLD and liver fibrosis.

### 3.2. Relationship between Dietary Intake and NAFLD or Liver Fibrosis

The Table 2 summarizes the association between quartiles of usual intake of nutrients with NAFLD in HIV participants. Considering the multivariate models, higher usual intake of total carbohydrates (highest quartile) was associated with lower odds for NAFLD [aOR = 0.44 (95% CI 0.24–0.8); *p* = 0.01] when compared to the lower intake range (reference quartile). Furthermore, participants with intermediate intake of fiber (third quartile), n6-PUFA (n6-poly-unsaturated FA) (second quartile), lauric FA (third quartile) had significantly lower odds for NAFLD when compared to the reference quartile, respectively aOR = 0.51 (95% CI 0.27–0.96); *p* = 0.04; aOR = 0.54 (95% CI 0.3–0.98); *p* = 0.04, and aOR = 0.42 (95% CI 0.22–0.78); *p* = 0.01. Additionally, a higher intake of myristoleic FA (fourth quartile) was a significant protective factor for NAFLD [aOR = 0.56 (95% CI 0.32–0.99), *p* = 0.05]. In contrast, participants with higher (fourth quartile) usual intake of total fat had higher odds for NAFLD compared to those with lower consumption [aOR = 1.91 (95% CI 1.06–3.44), *p* = 0.03].

The association between usual intake of nutrients in quartiles with occurrence of liver fibrosis in HIV mono-infected participants is summarized in Table 3. After adjustment for confounding factors, the usual intake of protein had only a statistical non-significant trend [aOR = 2.13 (95% CI 0.96–4.70); *p* = 0.06] to be associated with liver fibrosis. In multivariate models, participants with moderate usual intake of lauric FA [second quartile; aOR = 0.38 (0.18–0.80); *p* = 0.01], myristic FA [third quartile; aOR = 0.38 (0.17–0.89), *p* = 0.03], palmitoleic FA [third quartile; aOR = 0.40 (0.19–0.82); *p* = 0.01] and oleic FA [third quartile; aOR = 0.35 (0.16–0.79); *p* = 0.79] had lower risk of presence of liver fibrosis compared to those with low usual intake (lowest quartile) of these FAs. On the other hand, intermediate usual intake of n-6 PUFA (third quartile) was significantly associated with the presence of liver fibrosis compared to low intake [aOR = 2.45 (95% CI 1.12–5.32); *p* = 0.02].

## 4. Discussion

This study highlighted the association of dietary fat intake with the presence of NAFLD and/or fibrosis in PLWHA. To the best of our knowledge, this is the one of the first studies that has demonstrated the role of FA intake, and that high ingestion of total fat can increase the odds of NAFLD in PLWHA, independently of energy intake, age, sex and duration of c-ART. We demonstrated that participants with high usual intake of total fat had 91% more odds of having NAFLD.

A high-fat diet can be a trigger for liver fatty infiltration [29], might cause dysbiosis [30] and increase intestinal permeability leading to accumulation of triglycerides in hepatocytes contributing to NAFLD [31]. Our findings were aligned with a Korean study that showed a higher odds for NAFLD, determined by ultrasonography, in individuals with higher fat intake, quantified by food frequency questionnaire (FFQ) [32]. Similarly, high levels of Fatty Liver Index, a serological biomarker for detection of steatosis, were associated with a higher intake of total fat in a Dutch population [33]. In addition, a Brazilian cross-sectional study that assessed dietary intake in a limited sample of 96 participants with NAFLD using 24-HDR reported that most individuals consumed a higher total fat amount than recommended [14].

In contrast with previous publications, our study did not report association between total saturated fat intake and NAFLD. Instead, we described that moderate consumption of lauric FA was significantly associated with a lower odd of NAFLD and liver fibrosis in PLWHA. Lauric is a saturated medium-chain FA (MCFA) which is directly transported to the liver, where it is rapidly metabolized by β oxidation and also provokes a thermogenic response [34]. An experimental study reported that mice fed with lauric FA diet had lower obesity-related metabolic disorders and lower levels of plasma markers of liver function (alanine and aspartate aminotransferases) than mice fed with palmitic FA [35]. The present study also reported that participants with HIV who had moderated their consumption of myristic FA had less likely odds of having liver fibrosis compared to those with low intake. This might be explained by the fact that myristic, a saturated FA found in coconut and milk products, seems to be more rapidly metabolized (both β-oxidation and elongation) in hepatocytes [36].

The relationship between a high intake of monounsaturated fatty acid (MUFA) and improvement on lipid profile has been extensively described in previous studies that reported the benefits of the Mediterranean diet [10,37,38]. The present study reinforces this concept since we demonstrated that a moderate consumption of myristoleic FA, an MUFA, was a protective nutrient for NAFLD associated with lower odds of NAFLD. Additionally, moderate consumption of palmitoleic and oleic MUFAs were associated with a reduction of at least 60% in the odds for developing liver fibrosis. Several studies demonstrated that a diet rich in oleic acid can improve plasma lipid profile, inflammatory cytokines (INF-, IL-6), insulin sensitivity and macrophage infiltration, reducing histological features of NAFLD and liver fat [13,39,40]. Besides, previous studies reported that palmitoleic FA could impact glucose metabolism improving and/or preventing insulin resistance and type-2 diabetes [41].

Few studies have investigated the associations of n-6 PUFA intake with NAFLD. In our study, participants with a moderate consumption of n-6 PUFA had less likely NAFLD. This result is in agreement with a cross-sectional study that investigated the association of n-6 PUFA intake with NAFLD in adults using data from the National Health and Nutrition Examination Survey (NHANES). Those authors also used 24-HDR and demonstrated that n-6 PUFA intake was inversely associated with NAFLD [42]. In contrast, we reported that moderate consumption of n-6 PUFA increased the risk of liver fibrosis, probably related a pro-inflammatory activity [43]. These results were aligned with a study by Cortez-Pinto et al. which demonstrated that patients with biopsy-proven NASH had a significantly higher intake of n-6 PUFA and higher n6/n3 ratio, determined by FFQ, compared to controls [44].

We demonstrated that the moderate consumption of some fatty acids was associated with lower odds of NAFLD or liver fibrosis, but this was not observed in higher quartile. We suppose that the effect of dose—response might not be adequate for association of fatty acids with NAFLD or liver fibrosis because the moderate intake has a beneficial effect over excessive consumption.

We reported that a higher usual intake of total carbohydrate was associated with lower odds for NAFLD. Although the literature has shown that high intakes of dietary sugars have been associated with increased risk for NAFLD, there is no consensus about the effects of total carbohydrates on this liver disease [11]. Studies assessing nutrient intake and dietary patterns have showed that a high consumption of monosaccharides and disaccharides (fructose and sucrose) was positively associated with NAFLD [45,46]. We were unable to analyze the different subtypes of carbohydrates ingested, but we can suggest that our result reflects a high intake of polysaccharides, originating from beans and cereals, which are very common in Brazilian eating habits and are also sources of dietary fiber [47].

Another piece of evidence presented in this study revealed that a moderate consumption of dietary fiber was associated with lower odds for NAFLD, which remains in agreement with previous publications [46,48]. The benefits of dietary fiber have been extensively validated in overall metabolic health due to improvement of insulin sensitivity. Additionally, dietary fiber can prevent/control obesity through its effects on satiety, reducing the frequency of eating and the portion of food [49]. The fermentation of fiber, due to the interaction with gut microbes, can provide short-chain FA, key microbial metabolites that promote a protective and nourishing role for colonocytes, ensuring the preservation of the intestinal barrier and consequently protecting liver function [48].

The major limitations of our study are the cross-sectional study design and the lack of liver biopsy as the reference for the presence of NAFLD and/or liver fibrosis. Our study design does not allow us to conjecture any conclusions about the causality between dietary intake and incidence of NAFLD and/or fibrosis. In the present study, the presence of liver steatosis, for the definition of NAFLD, and the presence of fibrosis were defined using an extensive validated non-invasive method, such as transient electrography [50,51]. The same threshold of CAP measurement (≥248 dB/m) was used independent of the probe because LSM would be 1.5 to 2.0 kPa lower by the XL probe compared to M probe [52], but CAP seems to be similar in both probes [53]. A potential criticism would be the lack of physical activity assessment using validated questionnaires. We acknowledge that when informing the participant about the presence of liver steatosis and/or fibrosis during the clinical visit, it could affect in the second 24-HDR, due to significant changes in dietary habits. However, this source of bias was mitigated since we did not notice any important difference in food energy intake between first and second 24-HDRs. We are aware that 24-HDR is a self-reporting instrument for dietary intake assessment that might lead to underreporting, and to minimize this bias, we used the Automated Multiple-Pass method. Nevertheless, this is a practical and validated method that is considered the least biased to examine association between diet and disease and has been widely used in epidemiological and dietary monitoring studies [25]. Finally, we assume that two 24-HDRs might be insufficient to evaluate usual fat intake. However, we adjusted nutrients for total energy intake to minimize misreporting [53], as well as the potential variability on dietary intake using a well-established statistical method, such as MSM, to estimate usual intake [28]. The 24-HDR is a validated method that has been recommended as the least biased of the self-reporting instruments when compared to the other instruments such as FFQ and food record [25,54]. Alternative measurements which would be easier to implement in clinical practice are dietary screeners, which allow the assessment of aspects of the diet, such as specifics nutrients, rather than the total diet [55]. The lack of biochemical analysis of fatty acids is also a limitation of our study. Additionally, our study design hinders the evaluation of whether food intake can lead to higher prevalence of NAFLD in PLWHA compared to controls, since the PROSPEC-HIV was not set to include uninfected individuals. Studies comparing prevalence of NAFLD or food intake of PLWHA compared to controls (uninfected individuals) remain lacking in Brazil. However, the diet quality seems to be lower in PLWHA, and this population presents high prevalence of inappropriate food intake, despite the fact that PLWHA have undergone the same culture and influences as the general population [56,57]. The last point to highlight is the statistical methodological choices. We used the “Multivariate Nutrient Density Method” [58] due to the need to adjust the total energy consumption methodology (as mentioned earlier). This choice of statistical model highlighted the nutrients of interest (lipids) and avoided the discussion of diet, i.e., the influence of other nutrient intake on the correlation of lipid nutrients and their results. Thus, this choice can be considered a limitation of the study.

The main strengths of this study remain the dietary intake evaluation in a well-characterized large sample of people with HIV mono-infection, the quality methodology of data analysis and the use of DAG, supporting the choice of confounding variables. Clinical assessment, TE exams, bioelectrical impedances and blood samples were performed on the same day in the PROSPEC study. Additionally, all TE exams were performed for a single experimented operator in fasting patients, and blood analyses were performed in a centralized laboratory.

## 5. Conclusions

In conclusion, the current study showed that a higher usual intake of total fat increased the risk of NAFLD. Additionally, consumption of specific FAs was associated with lower and/or higher odds for presence of liver diseases in HIV mono-infected participants. These results reinforced the role of diet in the pathogenesis of NAFLD and/or liver fibrosis in PLWHA. Dietary assessment of total fat and FA could be incorporated into HIV care, and this strategy should be used as a tool for preventing NAFLD and fibrosis in PLWHA. Additionally, dietary supplementation of specific fatty acids, such as myristoleic FA, could be important in nutritional care of PLWHA.

## Figures and Tables

**Table 1:** Clinical and demographic characteristics of included participants with HIV mono-infection in INI/FIOCRUZ. Rio de Janeiro, Brazil.

Variables	All (*n* = 451)
Social and demographic	
Female sex ^a^	272 (60.3)
Age, years ^b^	45 (36–53)
Self-reported skin color ^a^	
White	214 (47.5)
Brown	139 (30.8)
Black	94 (20.8)
Others	4 (0.9)
Education ^a^ < 8 years of study	209 (46.4)
Comorbidities	
Diabetes mellitus ^a^	46 (10.2)
Hypertension ^a^	100 (22.2)
Dyslipidemia ^a^	78 (17.3)
Metabolic syndrome ^a^	150 (33.9)
Biochemistry	
ALT, IU/L ^b^	29 (23–43)
AST, IU/L ^b^	25 (20–33)
Alkaline phosphatase, IU/L ^b^	89 (70–111)
GGT, IU/L ^b^	45 (32–70)
Total cholesterol, mg/dL ^b^	185 (158–219)
LDL—cholesterol, mg/dL ^b^	112 (90–138)
HDL—cholesterol, mg/dL ^b^	43 (35–54)
Triglycerides, mg/dL ^b^	124 (84–171)
Fasting glucose, mg/dL ^b^	93 (88–100)
Insulin, um/L	11 (8–16)
HOMA-IR	3 (2–4)
Nutritional Status	
Body mass index, (kg/m^2^) ^b^	25 (23–29)
Lean [<25 Kg/m^2^] ^a^	207 (45.9)
Overweight [25–29.99 Kg/m^2^] ^a^	153 (33.9)
Obesity [≥30 Kg/m^2^] ^a^	91 (20.2)
Body fat, (%), by bioimpedance ^b^	30 (24–35)
Waist circumference, (cm) ^b^	87 (79–95)
HIV history and characteristics	
Duration of HIV infection, years ^b^	10 (5–17)
CD4+ T-lymphocyte count (cells/m^3^) ^b^	665 (421–881)
Detectable HIV RNA viral load (>40 cópias/mm^3^) ^a^	74 (16.1)
Current c-ART use ^a^	436 (96.7)
Duration of c-ART, years ^b^	7 (4–14)

Data expressed as *n* (%) ^a^ or median (IQR) ^b^. ALT, alanine transaminase; ART, antiretroviral therapy; AST, aspartate transaminase; BMI, body mass index; GGT, gamma-glutamyltransferase, high-density lipoprotein; HOMA-IR, homeostasis model assessment of insulin resistance; LDL, low-density lipoprotein; NAFLD, nonalcoholic fatty liver disease; waist circumference.

**Table 2:** Logistic multivariate model considering dietary intake and presence of nonalcoholic fatty liver disease (NAFLD) [CAP ≥ 248 dB/m] in participants with HIV mono-infection (*n* = 412)—INI/FIOCRUZ, Rio de Janeiro, Brazil.

	NAFLD	
Variables	Univariate Model	Multivariate Models
	OR (95% CI)	aOR (95% CI)
Energy; kcal		
Q1 < 1587.76	Reference	Reference
Q2 (1587.76–1952.72)	0.80 (0.46–1.39)	1.00 (0.50–2.00)
Q3 (1952.72–2299.45)	0.73 (0.42–1.28)	1.07 (0.43–2.68)
Q4 > 2299.45	0.68 (0.39–1.21)	1.43 (0.36–5.69)
Carbohydrate; % kcal		
Q1 < 49.07	Reference	Reference
Q2 (49.07–53.16)	0.62 (0.35–1.07)	0.56 (0.31–1.01)
Q3 (53.16–56.79)	0.56 (0.32–0.99)	0.56 (0.31–1.01)
Q4 > 56.79	0.48 (0.27–0.85)	0.44 (0.24–0.80)
Protein; % kcal		
Q1 < 14.49	Reference	Reference
Q2 (14.49–16.12)	1.19 (0.67–2.12)	0.99 (0.54–1.82)
Q3 (16.12–17.93)	1.37 (0.77–2.45)	1.18 (0.64–2.18)
Q4 > 17.93	1.71 (0.97–3.04)	1.41 (0.75–2.65)
Total fat; % kcal		
Q1 < 28.56	Reference	Reference
Q2 (28.56–31)	0.95 (0.53–1.68)	0.97 (0.53–1.76)
Q3 (31–34.38)	0.70 (0.39–1.26)	0.65 (0.35–1.21)
Q4 > 34.38	1.81 (1.04–3.17)	1.91 (1.06–3.44)
Fiber, density; g/1000 kcal		
Q1 < 8.47	Reference	Reference
Q2 (8.47–10.13)	0.87 (0.49–1.52)	0.71 (0.39–1.29)
Q3 (10.13–11.80)	0.68 (0.38–1.21)	0.51 (0.27–0.96)
Q4 > 11.80	1.13 (0.65–1.98)	0.88 (0.48–1.60)
Saturate fat; % kcal		
Q1 < 8.36	Reference	Reference
Q2 (8.36–9.59)	0.91 (0.51–1.61)	0.89 (0.49–1.62)
Q3 (9.59–10.77)	0.80 (0.45–1.41)	0.85 (0.46–1.55)
Q4 > 10.77	1.45 (0.83–2.54)	1.62 (0.9–2.92)
PUFA fat; % kcal		
Q1 < 5.87	Reference	Reference
Q2 (5.87–7.23)	0.63 (0.36–1.11)	0.57 (0.31–1.02)
Q3 (7.23–8.28)	0.68 (0.38–1.19)	0.60 (0.33–1.08)
Q4 > 8.28	0.77 (0.44–1.35)	0.70 (0.39–1.26)
MUFA fat; % kcal		
Q1 < 7.47	Reference	Reference
Q2 (7.47–8.38)	1.04 (0.59–1.83)	0.78 (0.43–1.43)
Q3 (8.38–9.48)	0.74 (0.42–1.34)	0.58 (0.31–1.07)
Q4 > 9.48	1.32 (0.75–2.31)	0.93 (0.49–1.76)
Trans FA; % kcal		
Q1 < 0.28	Reference	Reference
Q2 (0.28–0.37)	0.77 (0.44–1.37)	0.80 (0.44–1.46)
Q3 (0.37–0.46)	1.34 (0.77–2.35)	1.28 (0.71–2.31)
Q4 > 0.46	0.81 (0.46–1.43)	0.68 (0.37–1.25)
Cholesterol; % kcal		
Q1 < 0.1	Reference	Reference
Q2 (0.1–0.12)	1.33 (0.80–2.23)	1.25 (0.73–2.15)
Q3 (0.12–0.14)	0.99 (0.55–1.79)	0.84 (0.44–1.6)
Q4 > 0.14	1.14 (0.65–1.98)	0.96 (0.51–1.79)
*n*-6 PUFA; % kcal		
Q1 < 3.47	Reference	Reference
Q2 (3.47–4.34)	0.57 (0.32–1.01)	0.54 (0.30–0.98)
Q3 (4.34–5.28)	0.73 (0.41–1.27)	0.66 (0.37–1.20)
Q4 > 5.28	0.64 (0.36–1.13)	0.59 (0.32–1.08)
n-3 PUFA; % kcal		
Q1 < 0.41	Reference	Reference
Q2 (0.41–0.53)	0.75 (0.42–1.31)	0.71 (0.40–1.29)
Q3 (0.53–0.67)	0.72 (0.41–1.28)	0.65 (0.36–1.19)
Q4 > 0.67	0.89 (0.51–1.56)	0.76 (0.42–1.38)
Lauric FA (12:00); % kcal		
Q1 < 0.08	Reference	Reference
Q2 (0.08–0.13)	0.69 (0.40–1.19)	0.80 (0.45–1.40)
Q3 (0.13–0.18)	0.42 (0.23–0.77)	0.42 (0.22–0.78)
Q4 > 0.18	0.74 (0.44–1.27)	0.75 (0.43–1.32)
Myristic FA (14:00); % kcal		
Q1 < 2.36	Reference	Reference
Q2 (2.36–3.27)	1.23 (0.70–2.16)	1.25 (0.69–2.24)
Q3 (3.27–5.14)	1.00 (0.56–1.79)	1.09 (0.59–2.00)
Q4 > 5.14	1.34 (0.76–2.36)	1.26 (0.70–2.29)
Palmitic FA (16:00); % kcal		
Q1 < 2.58	Reference	Reference
Q2 (2.58–3.09)	1.23 (0.70–2.15)	1.30 (0.73–2.33)
Q3 (3.09–3.63)	1.30 (0.74–2.27)	1.20 (0.67–2.15)
Q4 > 3.63	0.89 (0.50–1.58)	0.82 (0.45–1.50)
Stearic FA (18:00); % kcal		
Q1 < 1.06	Reference	Reference
Q2 (1.06–1.3)	1.13 (0.65–1.99)	1.28 (0.72–2.30)
Q3 (1.3–1.57)	1.27 (0.73–2.22)	1.19 (0.66–2.14)
Q4 > 1.57	0.87 (0.49–1.55)	0.81 (0.44–1.49)
Arachidic FA (20:00); % kcal		
Q1 < 0.035	Reference	Reference
Q2 (0.035–0.04)	0.89 (0.53–1.51)	0.76 (0.44–1.33)
Q3 (0.04–0.05)	1.06 (0.61–1.83)	0.87 (0.49–1.55)
Q4 > 0.05	1.37 (0.66–2.83)	1.02 (0.47–2.21)
Myristoleic FA (14:1); % kcal		
Q1 < 0.02	Reference	Reference
Q2 (0.02–0.03)	0.78 (0.46–1.31)	0.72 (0.42–1.25)
Q3 (0.03–0.04)	0.53 (0.29–0.99)	0.63 (0.33–1.21)
Q4 > 0.04	0.70 (0.41–1.19)	0.56 (0.32–0.99)
Palmitoleic FA (16:1); % kcal		
Q1 < 0.17	Reference	Reference
Q2 (0.17–0.21)	0.80 (0.45–1.43)	0.80 (0.44–1.45)
Q3 (0.21–0.26)	1.27 (0.75–2.16)	1.06 (0.61–1.84)
Q4 > 0.26	1.08 (0.61–1.91)	0.87 (0.47–1.60)
Oleic FA (18:1); % kcal		
Q1 < 4.29	Reference	Reference
Q2 (4.29–5.09)	0.97 (0.55–1.71)	0.97 (0.54–1.75)
Q3 (5.09–5.94)	0.86 (0.49–1.52)	0.71 (0.39–1.29)
Q4 > 5.94	1.08 (0.61–1.89)	0.87 (0.47–1.58)
Linoleic FA (18:2-n6); % kcal		
Q1 < 3.45	Reference	Reference
Q2 (3.45–4.33)	0.61 (0.35–1.08)	0.59 (0.33–1.08)
Q3 (4.33–5.26)	0.75 (0.43–1.32)	0.70 (0.39–1.26)
Q4 > 5.26	0.66 (0.38–1.17)	0.63 (0.34–1.14)
Linolenic FA (18:3-n3); % kcal		
Q1 < 0.4	Reference	Reference
Q2 (0.4–0.51)	0.71 (0.40–1.26)	0.68 (0.38–1.24)
Q3 (0.51–0.63)	0.77 (0.44–1.34)	0.67 (0.37–1.21)
Q4 > 0.63	0.88 (0.50–1.55)	0.77 (0.43–1.40)
n6/n3 PUFA ratio; g		
Q1 < 7.45	Reference	Reference
Q2 (7.45–8.21)	0.61 (0.35–1.07)	0.59 (0.33–1.06)
Q3 (8.21–9.18)	0.66 (0.37–1.16)	0.69 (0.38–1.25)
Q4 > 9.18	0.84 (0.48–1.46)	0.96 (0.54–1.71)

Multivariate models adjusted by usual energy intake, age, gender and duration of c-ART. ART, antiretroviral therapy; CAP, controlled attenuation parameter; E%, energy percent; FA, fatty acid; g, gram; kcal, kilocalories; MUFA, mono-unsaturated FA; NAFLD, nonalcoholic fatty liver disease; PUFA, poly-unsaturated FA; Q, quartile.

**Table 3:** Logistic multivariate model considering dietary intake and presence of liver fibrosis (stage F ≥ 2) [LSM ≥ 7.1 kPa or ≥ 6.2 kPa with M or XL probe] in participants with HIV mono-infection (*n* = 443)—INI/FIOCRUZ. Rio de Janeiro, Brazil.

	Fibrosis	
Variables	Univariate Model	Multivariate Models
	OR [95%IC]	aOR [95%IC]
Energy; kcal		
Q1 < 1587.76	Reference	Reference
Q2 (1587.76–1952.72)	0.97 (0.49–1.89)	0.97 (0.43–2.20)
Q3 (1952.72–2299.45)	0.82 (0.41–1.64)	0.80 (0.26–2.46)
Q4 > 2299.45	0.50 (0.23–1.08)	0.48 (0.08–2.79)
Carbohydrate; % kcal		
Q1 < 49.07	Reference	Reference
Q2 (49.07–53.16)	1.33 (0.65–2.70)	1.37 (0.67–2.82)
Q3 (53.16–56.79)	1.25 (0.61–2.59)	1.33 (0.64–2.76)
Q4 > 56.79	0.99 (0.47–2.09)	0.97 (0.46–2.07)
Protein; % kcal		
Q1 < 14.49	Reference	Reference
Q2 (14.49–16.12)	1.94 (0.91–4.18)	1.75 (0.81–3.82)
Q3 (16.12–17.93)	1.11 (0.48–2.54)	0.97 (0.41–2.28)
Q4 > 17.93	2.61 (1.24–5.49)	2.13 (0.96–4.70)
Total fat; % kcal		
Q1 < 28.56	Reference	Reference
Q2 (28.56–31)	1.45 (0.75–2.80)	1.55 (0.79–3.05)
Q3 (31–34.38)	0.69 (0.33–1.46)	0.70 (0.33–1.49)
Q4 > 34.38	0.64 (0.30–1.37)	0.64 (0.30–1.39)
Fiber; g/1000 kcal		
Q1 < 8.47	Reference	Reference
Q2 (8.47–10.13)	0.77 (0.37–1.61)	0.65 (0.30–1.38)
Q3 (10.13–11.80)	0.83 (0.40–1.72)	0.69 (0.33–1.46)
Q4 > 11.80	1.21 (0.61–2.39)	0.95 (0.47–1.95)
Saturated fat; % kcal		
Q1 < 8.36	Reference	Reference
Q2 (8.36–9.59)	1.03 (0.52–2.02)	1.04 (0.53–2.08)
Q3 (9.59–10.77)	0.88 (0.44–1.77)	0.96 (0.47–1.96)
Q4 > 10.77	0.60 (0.28–1.28)	0.63 (0.29–1.36)
PUFA fat; % kcal		
Q1 < 5.87	Reference	Reference
Q2 (5.87–7.23)	1.67 (0.84–3.33)	1.61 (0.80–3.25)
Q3 (7.23–8.28)	0.94 (0.44–2.00)	0.89 (0.41–1.91)
Q4 > 8.28	1.03 (0.49–2.19)	0.96 (0.45–2.06)
MUFA fat; % kcal		
Q1 < 7.47	Reference	Reference
Q2 (7.47–8.38)	0.68 (0.33–1.41)	0.54 (0.26–1.14)
Q3 (8.38–9.48)	0.85 (0.42–1.70)	0.67 (0.33–1.38)
Q4 > 9.48	0.84 (0.42–1.68)	0.54 (0.25–1.17)
Trans FA; % kcal		
Q1 < 0.28	Reference	Reference
Q2 (0.28–0.37)	0.87 (0.40–1.90)	0.89 (0.41–1.96)
Q3 (0.37–0.46)	1.37 (0.65–2.88)	1.24 (0.58–2.65)
Q4 > 0.46	1.90 (0.94–3.84)	1.68 (0.82–3.45)
Cholesterol; % kcal		
Q1 < 0.1	Reference	Reference
Q2 (0.1–0.12)	1.20 (0.63–2.32)	1.10 (0.57–2.15)
Q3 (0.12–0.14)	1.05 (0.49–2.24)	0.87 (0.40–1.92)
Q4 > 0.14	1.29 (0.65–2.57)	1.03 (0.49–2.18)
n-6 PUFA; % kcal		
Q1 < 3.47	Reference	Reference
Q2 (3.47–4.34)	2.10 (0.96–4.59)	2.10 (0.95–4.63)
Q3 (4.34–5.28)	2.57 (1.19–5.54)	2.45 (1.12–5.32)
Q4 > 5.28	1.53 (0.68–3.47)	1.40 (0.61–3.21)
n-3 PUFA; % kcal		
Q1 < 0.41	Reference	Reference
Q2 (0.41–0.53)	0.66 (0.31–1.42)	0.64 (0.30–1.39)
Q3 (0.53–0.66)	1.34 (0.67–2.66)	1.27 (0.63–2.54)
Q4 > 0.655	1.08 (0.53–2.19)	0.94 (0.46–1.93)
Lauric FA (12:00); % kcal		
Q1 < 0.08	Reference	Reference
Q2 (0.08–0.13)	0.34 (0.16–0.72)	0.38 (0.18–0.80)
Q3 (0.13–0.18)	0.47 (0.23–0.98)	0.49 (0.24–1.02)
Q4 > 0.18	0.60 (0.31–1.16)	0.63 (0.32–1.22)
Myristic FA (14:00); % kcal		
Q1 < 2.36	Reference	Reference
Q2 (2.36–3.27)	0.97 (0.50–1.87)	0.98 (0.50–1.91)
Q3 (3.27–5.135)	0.36 (0.16–0.83)	0.38 (0.17–0.89)
Q4 > 5.135	0.85 (0.43–1.69)	0.80 (0.40–1.60)
Palmitic FA (16:00); % kcal		
Q1 < 2.58	Reference	Reference
Q2 (2.58–3.09)	0.71 (0.36–1.41)	0.73 (0.36–1.46)
Q3 (3.09–3.625)	0.66 (0.32–1.34)	0.62 (0.30–1.28)
Q4 > 3.625	0.71 (0.36–1.41)	0.64 (0.32–1.30)
Stearic FA (18:00); % kcal		
Q1 < 1.06	Reference	Reference
Q2 (1.06–1.3)	0.76 (0.38–1.51)	0.82 (0.41–1.64)
Q3 (1.3–1.57)	0.50 (0.23–1.07)	0.48 (0.22–1.04)
Q4 > 1.57	0.95 (0.48–1.87)	0.91 (0.46–1.80)
Arachidic FA (20:00); % kcal		
Q1 < 0.035	Reference	Reference
Q2 (0.035–0.04)	0.93 (0.48–1.81)	0.81 (0.41–1.6)
Q3 (0.04–0.05)	1.00 (0.50–1.98)	0.83 (0.41–1.69)
Q4 > 0.05	1.29 (0.53–3.14)	0.98 (0.39–2.46)
Myristoleic FA (14:1); % kcal		
Q1 < 0.02	Reference	Reference
Q2 (0.02–0.03)	0.65 (0.34–1.27)	0.63 (0.32–1.24)
Q3 (0.03–0.04)	0.51 (0.23–1.14)	0.59 (0.26–1.34)
Q4 > 0.04	0.71 (0.37–1.38)	0.65 (0.33–1.27)
Palmitoleic FA (16:1); % kcal		
Q1 < 0.17	Reference	Reference
Q2 (0.17–0.21)	0.78 (0.40–1.52)	0.80 (0.40–1.58)
Q3 (0.21–0.26)	0.46 (0.23–0.95)	0.40 (0.19–0.82)
Q4 > 0.26	0.68 (0.34–1.35)	0.52 (0.25–1.09)
Oleic FA (18:1); % kcal		
Q1 < 4.29	Reference	Reference
Q2 (4.29–5.09)	0.74 (0.37–1.46)	0.71 (0.36–1.43)
Q3 (5.09–5.94)	0.40 (0.18–0.88)	0.35 (0.16–0.79)
Q4 > 5.94	0.89 (0.46–1.73)	0.70 (0.35–1.41)
Linoleic FA (18:2-n6); % kcal		
Q1 < 3.45	Reference	Reference
Q2 (3.45–4.33)	1.61 (0.75–3.44)	1.63 (0.75–3.51)
Q3 (4.33–5.23)	2.17 (1.04–4.53)	2.08 (0.99–4.38)
Q4 > 5.23	1.30 (0.59–2.84)	1.19 (0.54–2.64)
Linolenic FA (18:3-n3); % kcal		
Q1 < 0.4	Reference	Reference
Q2 (0.4–0.51)	0.68 (0.31–1.46)	0.67 (0.31–1.45)
Q3 (0.51–0.63)	1.32 (0.67–2.61)	1.25 (0.63–2.49)
Q4 > 0.63	1.04 (0.51–2.14)	0.92 (0.44–1.91)
n6/n3 PUFA ratio, g		
Q1 < 7.445	Reference	Reference
Q2 (7.445–8.21)	1.69 (0.82–3.51)	1.69 (0.81–3.52)
Q3 (8.21–9.18)	1.57 (0.75–3.30)	1.62 (0.77–3.44)
Q4 > 9.18	1.20 (0.56–2.60)	1.27 (0.58–2.78)

Multivariate models adjusted by usual energy intake, age, gender and duration of c-ART. ART, antiretroviral therapy; E%, energy percent; kcal, kilocalories; LSM, liver stiffness measurement; MUFA, mono-unsaturated FA; NAFLD, nonalcoholic fatty liver disease; PUFA, poly-unsaturated FA; Q, quartile.

## Data Availability

Not applicable.

## Supplementary Materials

The following are available online at https://www.mdpi.com/article/10.3390/nu13103462/s1, Table S1: Clinical and demographic characteristics of participants with HIV mono-infection in INI/FIOCRUZ and nonalcoholic fatty liver disease (NAFLD) [CAP ≥ 248 dB/m; *n* = 152, prevalence = 37%], Rio de Janeiro, Brazil, Table S2: Clinical and demographic characteristics of participants with HIV mono-infection in INI/FIOCRUZ and significant liver fibrosis (stage F ≥ 2) [LSM ≥ 7.1 kPa or ≥6.2 kPa with M or XL probe; *n* = 72, prevalence = 16%], Rio de Janeiro, Brazil.

## Abbreviations

ALT, alanine aminotransferase; ART, antiretroviral therapy; AST, aspartate aminotransferase; AUDIT, Alcohol Use Disorders Identification Test; BMI, body mass index; CAP, controlled attenuation parameter; CI, confidence interval; E%, energy percent; FA, fatty acid; FFQ, frequency food questionnaire; GGT, gamma-glutamyltransferase; 24-HDR, 24 h dietary recall; HDL, high-density cholesterol; IQR, interquartile range; LDL, low-density cholesterol; LSM, liver stiffness measurement; MUFA, mono-unsaturated FA; nonalcoholic NAFLD, nonalcoholic fatty liver disease; NASH, nonalcoholic steatohepatitis; OR, odds ratio; PUFA, poly-unsaturated FA; Q, quartile; ULN, upper limit of normal; WHO, World Health Organization.
