# Supplementary material for: Relationship between Dietary Fatty Acid Intake with Nonalcoholic Fatty Liver Disease and Liver Fibrosis in People with HIV
Source: Nutrients. 2021 Sep 29;13(10):3462. doi: 10.3390/nu13103462 (PMC8539489; doi:10.3390/nu13103462)
Supplement: Supplementary file 1 [file nutrients-13-03462-s001.zip › nutrients-1310006-supplementary.pdf]

Supplementary Material

Relationship between dietary FA intake with NAFLD and/or liver fibrosis in people with HIV mono-infection

Supplementary Table S1. Clinical and demographic characteristics of participants with HIV mono-infection and non-alcoholic fatty liver disease (NAFLD) [CAP ≥ 248 dB/m; n=152, prevalence= 37%] included in the analysis in INI/FIOCRUZ. Rio de Janeiro, Brazil

		**NAFLD (n=152)**
**Social and demographic**		
Female sex[a]		87 (57.2)
Age, years[b]		50 (41 - 55)
Self-reported skin color[a]		
	White	82 (53.9)
	Brown	42 (27.6)
	Black	27 (17.8)
	Others	1 (0.7)
Education[a] <8 years of study		74 (48.7)
**Biochemistry**		
ALT, IU/L[b]		34 (25 - 50)
AST, IU/L[b]		25 (20 - 34)
Alkaline phosphatase, IU/L[b]		87 (68 - 110)
GGT, IU/L[b]		51 (37 - 85)
Total cholesterol, mg/dL[b]		196 (171 - 222)
LDL - cholesterol, mg/dL[b]		116 (91 - 142)
HDL - cholesterol, mg/dL[b]		41 (33 - 50)
Triglycerides, mg/dL[b]		165 (112 - 243)
Fasting glucose, mg/dL[b]		98 (89 - 106)
Insulin, um/L		15 (10 - 20)
HOMA-IR		4 (2 - 5)
**Nutritional Status**		
BMI(kg/m^2) [b]		29 (26 - 32)
BMI(kg/m^2) [a]		
	Lean [< 25 Kg/m^2][a]	27 (17.8)
	Overweight [25 – 29,99 Kg/m^2] [a]	65 (42.8)
	Obesity [≥ 30 Kg/m^2][a]	60 (39.5)
Body fat, (%) by bioimpedance[b]		32 (27 - 37)
**Comorbidities**		
Diabetes mellitus[a]		34 (22.4)
Hypertension[a]		52 (34.2)
Dyslipidaemia[a]		44 (28.9)
Metabolic syndrome[a]		87 (58.8)
**HIV infection and ART**		
Duration of HIV, years[b]		12 (7 - 19)
CD4+ T-lymfocyte count (cells/m^3)[b]		708 (524 - 935)
Current ART use[a]		147 (96.7)

Duration of ART, years[b]	10 (4 - 16)

Data expressed as n (%) [a] or median (IQR) [b]. ALT, alanine transaminase; ART, antiretroviral therapy; AST, aspartate transaminase; BMI, body mass index; GGT, gamma-glutamyltransferase, high-density lipoprotein;HOMA-IR, homeostasis model assessment of insulin resistance; LDL, low-density lipoprotein; NAFLD, non-alcoholic fatty liver disease.

Supplementary Table S2 Clinical and demographic characteristics of participants with HIV mono-infection and significant liver fibrosis (stage F≥2) [LSM ≥ 7.1 kPa or ≥ 6.2 kPa with M or XL probe; n=72, prevalence= 16%] included in the analysis in INI/FIOCRUZ. Rio de Janeiro, Brazil

		**Liver fibrosis n=72**
**Social and demographic**		
Female sex[a]		42 (58.3)
Age, years[b]		47 (41 - 54)
Self-reported skin color[a]		
	White	33 (45.8)
	Brown	20 (27.8)
	Black	18 (25)
	Outros	1 (1.4)
Education[a] <8 years of study		37 (51.4)
**Biochemistry**		
ALT, IU/L[b]		36 (25 - 56)
AST, IU/L[b]		29 (22 - 41)
Alkaline phosphatase, IU/L[b]		86 (68 - 108)
GGT, IU/L[b]		54 (36 - 111)
Total cholesterol, mg/dL[b]		176 (153 - 203)
LDL - cholesterol, mg/dL[b]		102 (83 - 125)
HDL - cholesterol, mg/dL[b]		42 (35 - 52)
Triglycerides, mg/dL[b]		140 (80 - 187)
Fasting glucose, mg/dL[b]		95 (90 - 103)
Insulin, um/L		12 (7 - 20)
Homa-IR		3 (2 - 5)
**Nutritional Status**		
BMI(kg/m^2) [b]		29 (24 - 33)
BMI(kg/m^2) [a]		
	Lean [< 25 Kg/m^2][a]	25 (34.7)
	Overweight [25 – 29,99 Kg/m^2] [a]	18 (25)
	Obesity [≥ 30 Kg/m^2][a]	29 (40.3)
Body fat, (%) by bioimpedance[b]		32 (24 - 38)
waist circunference (cm)		91 (82 - 104)
**Comorbidities**		
Diabetes mellitus[a]		16 (22.2)
Hypertension[a]		29 (40.3)
Dyslipidaemia[a]		17 (23.6)
Metabolic syndrome[a]		32 (45.1)
**HIV infection and ART**		
Duration of HIV, years[b]		11 (4 - 16)
CD4+ T-lymfocyte count (cells/m^3)[b]		648 (361 - 842)
CD4+ T-lymfocyte count (<200cells/m^3)[a]		5 (7.1)
Current ART use[a]		68 (94.4)
Duration of ART, years[b]		10 (3 - 15)

Data expressed as n (%) [a] or median (IQR) [b]. ALT, alanine transaminase; ART, antiretroviral therapy; AST, aspartate transaminase; BMI, body mass index; GGT, gamma-glutamyltransferase, high-density lipoprotein;HOMA-IR, homeostasis model assessment of insulin resistance; LDL, low-density lipoprotein; NAFLD, non-alcoholic fatty liver disease.
